# Risk of cervical abnormality after age 50 in women with previously negative smears

**DOI:** 10.1038/sj.bjc.6605069

**Published:** 2009-05-05

**Authors:** R G Blanks, S M Moss, S Addou, D A Coleman, A J Swerdlow

**Affiliations:** 1 Cancer Screening Evaluation Unit, Sir Richard Doll Building, Institute of Cancer Research, 15 Cotswold Road, Sutton, Surrey SM2 5NG, UK

**Keywords:** screening, cervical cancer, age

## Abstract

There is discussion over the benefit of continuing cervical screening in women over the age of 50 with a history of negative cytology. We aimed to determine the risk of abnormal cytology in such women. Screening history data from 1985 to 2003 were obtained for a cohort of 2 million women from the NHS cervical screening programme from four Health Authorities in England. The 57 651 women in the cohort who reached age 40 between 1 January 1985 and 31 December 1990 and had at least one routine or opportunistic smear between ages 50 and 54 were included in the analysis. Exposure groups (negative cytology history, negative but including inadequate smears, and positive history) were defined on the basis of screening histories from ages 40 to 49. Sixty-four percent (134/206) (95% CI: 57–71%) of the moderate dyskaryosis or worse lesions at ages over 50 were detected from women in the negative smear history group. After allowance for time since last negative smear, the relative risk for the first primary smear over the age of 50 having moderate dyskaryosis or worse decreased from 0.60 (95% CI: 0.41–0.84) for two negative smear episodes to 0.25 (95% CI: 0.10–0.56) for four negative smear episodes, compared with the positive history group. If screening were discontinued for all women over 50 with a negative history, the majority of cytological abnormalities now being detected at these ages that lead directly to referral to colposcopy would be missed.

There has been much discussion since the early 1990's about the benefit of continuing cervical screening in women over the age 50 years with a history of repeated negative cytology. It has been suggested that such women are at very low risk of having pre-invasive lesions and therefore of developing invasive cancer, and that ceasing screening in this group could alleviate anxiety and enable better allocation of resources (Van Wijngaarden and Duncan, 1993; Cruickshank *et al*, 1997).

Cervical screening aims to prevent the occurrence of invasive cancer by detecting pre-invasive lesions (CIN 1, 2 and 3). The prevalence of pre-invasive cervical lesions decreases with age and women screened over the age of 50 have a considerably lower risk of having abnormal cytology results. The risk of mild or worse dyskaryosis varies from around 10% for women in their early twenties to only around 1% for women over 50 years (Cervical screening programme, England 2007–08 statistical bulletin). The rate of progression of most lesions destined to become invasive cancer is generally considered to be slow, and one might therefore anticipate that a negative smear history before the age of 50 would considerably reduce the risk of positive cytology or histology in women aged 50–64 years (the upper age of invitation to screening in the UK), who have been regularly screened. In the 1990's it was suggested, based on evidence from the Tayside area of Scotland, that women over the age of 50 years with an adequate history of negative results on smear testing every 3 years may be safely discharged from further screening (Van Wijngaarden and Duncan, 1993). There have been few studies to test this, however (Cruickshank *et al*, 1997; Flannelly *et al*, 2004; Armaroli *et al*, 2008), and the only large study directly considering the question had relatively short screening histories (Flannelly *et al*, 2004).

We have therefore undertaken a large cohort study to investigate the risk of a positive primary smear result at the first routine recall episode after the age of 50 years in women with a negative smear history at ages 40–49 years compared with women with other histories, and how the number of negative smears relates to this risk.

## Methods

We assembled a population-based cohort of 2 million women from four health authorities (two areas invited 3 yearly and two other areas 5 yearly) in England with cervical screening histories covering the period 1 January 1985 to around March 2004 (the exact date dependent on the area of residence), using information obtained from the national computerised call-recall system. Women within this cohort with dates of birth between 1 January 1945 and 31 December 1950 and still resident and eligible in the same areas for invitation to screening at March 2004 have been included in the current analysis. These women reached age of 40 years between 1 January 1985 and 31 December 1990 (around the start of the national computerised call-recall system circa 1988) and we have details on the computerised system of 10 years of screening history between ages of 40 and 49 years and a minimum of 3 years follow-up after reaching age of 50 years.

For analysis, we divided the screening histories into ‘episodes’ starting with a routine or opportunistic (‘primary’) smear and ending with a (‘closing’) smear that returned the woman back to routine recall or cessation of screening (e.g., because of a hysterectomy). For most episodes the primary smear was negative with an action of return to routine recall, and therefore the primary smear was also the closing smear. We included in our analysis women in the cohort with a primary smear between the ages of 50 and 54 years; these are therefore routine or opportunistic smears and not repeat or follow-up tests. The cohort and our use of episodes is described in detail in an earlier paper (Blanks *et al*, 2007).

We allocated women to three groups for analysis on the basis of their screening history between ages of 40 and 49 years. ‘Negative’ history was defined as at least two episodes, all of which were single smear episodes with a negative result and an action of return to routine recall. ‘Inadequate’ history was defined as one or more episodes that included an inadequate smear result, but no abnormal cytology results. ‘Positive’ history was defined as one or more episodes that included a smear with results of borderline abnormal cytology or worse. Women with smear histories consisting of only one negative episode were not included in the study.

The main outcome measure was the prevalence of cytological disease in the primary smear from the first episode starting after the age of 50 years, with the additional criterion that the episode must have occurred before age of 55 years. We analysed the prevalence ratio (also alluded to in the text as relative risk) of cytological disease in this first primary smear over 50 years in the ‘negative’ compared with the ‘positive’ group and the effect of increasing numbers of negative episodes before age of 50 years. A logistic regression analysis was used to determine any confounding effect of duration between the last smear under age 50 years and the first smear after age of 50 years, because the length of this interval affects the risk of abnormality after age of 50 years, and could be related to whether the earlier smear was positive, inadequate or negative and the frequency of past screening. The exposure of two, three or four negative smear episodes before age of 50 years was entered into the model as a categorical variable. There were four final models with outcomes of, respectively, borderline disease or worse; mild dyskaryosis or worse; moderate dyskaryosis or worse and severe dyskaryosis or worse. The outcomes of the models were odds ratios, but these can be treated as relative risks as the vast majority of women (with adequate smear results) will have negative results and therefore positive cytological disease outcomes can be considered rare.

In addition, the probability of referral to colposcopy during the first episode after age of 50 years was calculated for ‘negative’, ‘positive’ and ‘inadequate’ groups and the prevalence ratio of referral to colposcopy for the ‘negative’ and ‘inadequate’ groups relative to the ‘positive’ group was calculated. All statistical analyses were conducted using STATA version 8 (StataCorp, College Station, TX, USA).

To examine the effects of 3- and 5-yearly screening policy, we examined the probability of women with negative smear histories of either two or three episodes between the ages of 40 and 49 years having a primary smear result of mild dyskaryosis or worse at their first episode after age of 50 years.

## Results

There were 71 283 women in the three exposure groups defined by their screening history between ages of 40 and 49 years, of whom 57 671 (81%) also had a primary smear between the ages of 50 and 54 years and were therefore included in the analysis. Of these, 42 124 were in the ‘negative’ group, 7056 in the ‘inadequate’ group and 8471 in the ‘positive’ group. The group of women included in the study reached age of 50 years between 1 January 1995 and 31 December 2000 and the study outcome primary smears occurred between 9 January 1995 and 17 March 2004. The mean age for the outcome primary smear was 51.4 years (s.d. 1.2 years).

Table 1 shows details of the primary smear results for the first screening episode over the age of 50 years for the three study groups. The ‘negative’ group had a higher proportion of negative smear results (91.7%) from the first smear over 50 years compared with the ‘inadequate’ (88.1%) and ‘positive’ (89.4%) groups. The ‘inadequate’ group, however, had a notably higher proportion of inadequate smear results, suggesting that an earlier inadequate smear history is predictive of future inadequate smears. Table 1 also shows the proportion of adequate smears with moderate dyskaryosis or worse for all three groups, with 95% confidence limits. Of 209 moderate dyskaryosis or worse smear results, 134 (64% (95% CI: 57–71%)), were from the negative history group.

Table 2 shows an analysis according to whether women had two, three or four negative smear episodes between ages of 40 and 49 years. For moderate dyskaryosis or worse, the risk compared with the ‘positive’ group for two negative episodes was 0.60 (95% CI: 0.41–0.84), for three negative episodes 0.47 (95% CI: 0.32–0.71) and for four negative episodes 0.25 (95% CI: 0.10–0.56). It is relatively uncommon for a woman to have had four routine episodes between 40 and 49 (usually occurring when a women has been screened every 3 years and her first smear included in the study was at exactly the age of 40 years) and this group is therefore small and the confidence limits wide.

The risks of cytological abnormality at the first primary smear over age of 50 years in relation to number of negative smears at ages of 40–49 years, relative to the ‘positive’ group and adjusting for the effects of time since last smear, are shown in Table 3. The relative risks of moderate dyskaryosis or worse for two, three and four negative episodes were 0.58 (95% CI: 0.39–0.84), 0.49 (95% CI: 0.33–0.73) and 0.27 (95% CI: 0.12–0.61), respectively. The results are similar to the unadjusted values and confirm that after allowing for time since last smear there is a continuing reduction in risk associated with increasing numbers of negative smear episodes and suggestive evidence that the reduction may be greatest for higher levels of disease (i.e., those most likely to progress to invasive cancer).

Cancer registry information obtained by the CSEU has included information on cases of CIN 3 in the study subjects, but the data were not sufficiently complete or timely to be analysed statistically as an outcome measure in this study. However, even in these incomplete data there were 19 CIN 3 registrations and six invasive cancer registrations from women in the ‘negative’ group (including two invasive cancer registrations occurring in women with four negative smear episodes between ages of 40 and 49 years), showing clearly that a negative smear history between ages of 40 and 49 years does not preclude the occurrence of invasive cancer after age of 50 years. We have checked the screening histories of the six women with invasive cancers detected from the first smear after the age of 50 years after negative smear histories. The smear test results and the actions taken as a consequence of the smears are consistent with the detection of such an abnormality, and hence we are confident that the recorded abnormalities are not data entry errors.

The percentage of women referred to colposcopy (Table 4) for the ‘negative’ group was 1.18% compared with 2.35% for the ‘positive group’, a relative risk of 0.48 (95% CI: 0.41–0.57).

The negative smear history group can be divided into women in 3- or 5-yearly screening policy areas with two or three negative episodes between ages 40 and 49 years. The percentage adequate smears with mild or worse cytology was 0.67% (95% CI: 0.48–0.91) for two negative episodes and 0.61% (95% CI: 0.48–0.76) for three negative episodes. For women in 5 yearly screening areas these figures were 1.09% (95% CI: 0.91–1.30) and 0.80% (95% CI: 0.58–1.08), respectively. As might be expected, the probability of mild or worse cytology is higher in 5-yearly than 3-yearly screening policy areas.

## Discussion

Our results show that women over the age of 50 years with a history of a minimum of two negative screening episodes between the ages of 40 and 49 years, have a lower risk of cytological disease found at screening than women with a ‘positive’ history. The relative risk for borderline or worse disease at ages over 50 years varied from 0.67 for women with two negative episodes to 0.52 for women with four negative episodes compared with women with a positive disease history. For severe dyskaryosis or worse the relative risk decreased from 0.49 for two negative episodes to 0.13 for four negative episodes. There is therefore evidence that the risk of higher levels of cytological disease is reduced more with increasing numbers of earlier negative smear episodes than is the case for lower levels of cytological disease. Of the 209 moderate dyskaryosis or worse smear results 134 (64% (95% CI: 57–71%)) were from the negative history group and about 75–80% of these women would be expected to have histological outcomes of CIN 2 or worse, based on the reported positive predictive value of moderate dyskaryosis or worse for histology of CIN 2 or worse by the local laboratories (Cervical Screening Programme bulletin, 2000–01). The negative history group were therefore clearly not a group for whom withdrawal of screening would not be material in public health policy terms.

Some of the women in the cohort will have received 5-yearly screening invitations and some 3-yearly invitations, although in practice because of the use of ‘opportunistic’ smear tests many women in 5-year policy areas will have had screening at a shorter interval than this (Flannelly *et al*, 2004). Women receiving 5 yearly invitations were more likely to have had only two invitations. The 3-year policy areas may be considered more important to current UK screening policy as they reflect the current screening policy more closely. The results suggest a marginally reduced risk of mild dyskaryosis or worse with number of negative episodes if 3-year policy areas only are considered, but the conclusion remains that if screening were discontinued in women with negative smear histories an appreciable number of abnormalities would be missed.

The four Health Authority areas included in the study were chosen because they had demographically similar populations to each other and therefore might have similar disease risk, but had different screening policies. Note that the screening history of women in our cohort (if any) before 1985 is unknown and this is why our study is limited to using only the screening history of women between ages of 40 and 49 years to determine ‘exposure’ groups. The computerised national call-recall system started in 1988 and there is some possibility that records of a very few smears from 1985 to 1987 may be missing from the files we used for these years, but too few to have affected the results materially.

In general the risk of pre-invasive disease in women aged over 50 years is much lower than in younger women and therefore women over the age of 50 years with a negative smear history (and particularly with at least four negative smear episodes) are the lowest identifiable risk group in our cohort. Nevertheless, our results, suggest that if screening were discontinued for women over 50 years with past negative smears, appreciable morbidity would be missed. Selectively stopping screening at age 50 years for just those with four serial negative smears would result in far less missed morbidity, but with lower cost savings because the number of subjects with four serial negatives was only one-eighth of the number with two or three serial negatives. It is possible that taking account of negative smears over a longer age-span than 40–49 years, or considering cessation of screening at age of 55 years, would identify a group with clearer cost benefit from cessation of screening, but the NHS screening programme data do not, yet, run for a long enough period to assess this. When longer follow-up becomes available, with the passage of time, we will investigate risk of disease in women with a negative history for the 15-year period between the ages of 35–49 years, and outcomes for ages older than 55 years.

The cohort is formed from residents of Health Authority areas in the South of England, which are likely to have a lower risk of cervical cancer than the English average (Swerdlow and dos Santos Silva, 1993). This is borne out by the percentage of adequate smears having mild or worse dyskaryosis being 1.4% for women aged of 50–54 years in England (Cervical screening programme, England 2007–08 statistical bulletin), but only 0.9% in our cohort sample. This suggests that the number of abnormalities being missed would be even greater in the national screening programme as a whole than in our cohort.

There have been a number of earlier investigations into the risk of cervical disease in women aged over 50 years with negative smear histories. Van Wijngaarden and Duncan (1993) reported that of 26 women with micro-invasive and invasive cancer registered in the Tayside area of Scotland at ages over 50 years, none had had two or more serial negative 3-yearly smears. They also reported that newly occurring cases of CIN were not seen in women over 50 years who had been screened every 3 years. They, therefore, suggested that women should cease screening at age 50 years if they had had three previous negative smears. Cruickshank *et al* (1997) reported that among ∼9000 women regularly screened every 3 years before the age of 50 years, one case of CIN 3 and one case of invasive cancer were detected between 50 and 60, giving a low disease rate, but with very wide confidence limits. Flannelly *et al* (2004), by contrast, reported that 1.8% of 36 512 women with a negative smear history still showed subsequent dyskaryosis over the age of 50. The period of screening considered was relatively short, however; both the negative smears and the smear over age of 50 years had to have occurred within an 8-year period.

Other studies have investigated smear outcomes after negative smears, but not specifically the question put forward by Van Wijngaarden (Van Wijngaarden and Duncan, 1993), that is, risks of abnormality detected at smears after the age of 50 years in women with a negative history before that age. Armaroli *et al* (2008) reported that the cumulative risk of CIN 2 or worse was at least eightfold higher in women aged less than 50 years after one earlier negative test than in women over 50 years with four serial negative tests at any age. Armaroli *et al* (2008) also found, as we did, that risks of abnormality decreased with increasing numbers of negative smears – a finding also noted in a Canadian study (Coldman *et al*, 2005).

Consideration of whether screening should cease beyond the age of 50 years for those with a past negative history needs to take account of both the potential benefits and harms of screening. The potential harm associated with screening includes unnecessary treatment from over-diagnosis and increased anxiety as well as the cost of screening. It has been suggested that HPV testing could be beneficial in identifying the small proportion of women still at risk after the age of 50 years (Cruickshank *et al*, 2002). Sherlaw-Johnson *et al* (1999) used mathematical modelling to study the effects of withdrawing women at the age of 50 years from screening, who had a recent history of negative results, or where the last smear was negative and they tested negative for high-risk HPV. They concluded that early withdrawal of women from the programme could give resource savings of up to 25% for cytology and 18% for colposcopy at the cost of an increased risk for cervical cancer of up to two cases per 100 000 women per year.

In conclusion, from our data the risk of pre-invasive cervical disease at the age of 50 years in women with a history of multiple negative smears between ages 40 and 49 years was moderately reduced. At present, the NHS cervical screening programme using the national call-recall system (which started in 1988) can only determine a negative history over a limited period of time, and the same was therefore true for our study. Our data give evidence that the risk of abnormality, and particularly the risk of more severe pre-invasive lesions, may decline with increasing numbers of earlier negative smear episodes. Longer follow-up of the cohort will enable the outcome of women with more extensive negative histories to be studied, to determine if there are potentially very low risk groups for whom further screening may not be the best use of resources.

## Figures and Tables

**Table 1:** Result of the first primary smear test after age of 50 years by outcome of screening at ages of 40–49 years

	**Screening history between ages 40 and 49 years**					
**Reported outcome of first primary**	**Negative^a^**		**Inadequate^b^**		**Positive^c^**	
**smear after age of 50 years**	**Number**	**%**	**Number**	**%**	**Number**	**%**
Negative	38 633	91.71	6218	88.12	7569	89.35
Inadequate	2283	5.42	607	8.60	503	5.94
Borderline	895	2.12	172	2.44	287	3.39
Mild dyskaryosis	179	0.42	36	0.51	60	0.71
Moderate dyskaryosis	69	0.16	11	0.16	22	0.26
Severe dyskaryosis	43	0.10	6	0.09	20	0.24
Severe dyskaryosis-query invasive	3	0.01	0	0.00	1	0.01
Glandular neoplasia	19	0.05	6	0.09	9	0.11
Total	42 124		7056		8471	
						
Median no. of primary smears between ages of 40 and 49 years	3		3		3	
Moderate dyskaryosis or worse (% of adequate smears and 95% CI)	134 (0.34% 95% CI: 0.28–0.40)		23 (0.36% 95% CI: 0.22–0.53)		52 (0.65% 95% CI: 0.48–0.85)	
						

a Negative: at least two episodes, all of which were single smear episodes with a negative result and an action of return to routine recall.

b Inadequate: one or more episodes that included an inadequate smear result, but no abnormal cytology results.

c Positive: one or more episodes that included a smear with results of borderline cytology or worse.

**Table 2:** Prevalence ratio (relative risk) of various levels of abnormal primary smear at the first smear over age of 50 years in relation to smear histories between ages of 40 and 49 years

**Screening history between 40 and 49 years**	**Adequate primary smears**	**Borderline or worse No. PR (95% CI)**		**Mild or worse No. PR (95% CI)**		**Moderate or worse No. PR (95% CI)**		**Severe or worse No. PR (95% CI)**	
Negative	**39 841**	**1208**	**0.61 (0.54–0.68)** ^***^	**313**	**0.56 (0.45–0.79)** ^***^	**134**	**0.52 (0.37–0.72)** ^***^	**65**	**0.43 (0.28–0.69** ^***^
Two negative episodes	17 250	621	0.72 (0.63–0.82)^***^	163	0.67 (0.53–0.86)^***^	67	0.60 (0.41–0.84)^**^	34	0.52 (0.31–0.89)^*^
Three negative episodes	17 746	455	0.51 (0.45–0.59)^***^	118	0.47 (0.36–0.62)^***^	55	0.47 (0.32–0.71)^***^	26	0.39 (0.22–0.68)^***^
Four negative episodes^a^	4211	103	0.49 (0.39–0.61)^***^	22	0.37 (0.22–0.59)^***^	7	0.25 (0.10–0.56)^***^	2	0.13 (0.01–0.50)^***^
Inadequate	**6449**	**231**	**0.72 (0.61–0.84)** ^***^	**59**	**0.65 (0.47–0.90)** ^**^	**23**	**0.55 (0.32–0.91)** ^*^	**12**	**0.49 (0.23–0.99)** ^*^
Positive	**7968**	**399**	**1.00**	**112**	**1.00**	**52**	**1.00**	**30**	**1.00**

PR=Prevalence ratio, 95% CI=95% confidence interval.

^*^P<0.05, ^**^P<0.01, ^***^P<0.001.

a In addition there were 634 women with 5+ negative smear episodes not included in sub-group analysis because of small numbers and because the women are less likely to be representative of normal screening histories as a history of five or more episodes over a 10-year period is not consistent with routine 3 (or 5) yearly screening.

**Table 3:** Odds ratios (relative risks) for various levels of abnormality for the first smear after the age of 50 years by number of negative smear episodes between the ages of 40 and 49 years, after allowance for time since last smear

**Exposure group**	**Borderline or worse OR (95% CI)**	**Mild dyskaryosis or worse OR (95% CI)**	**Moderate dyskaryosis or worse OR (95% CI)**	**Severe dyskaryosis or worse OR (95% CI)**
Positive	1.00	1.00	1.00	1.00
Two negative episodes	0.67 (0.59–0.77)^***^	0.62 (0.48–0.80)^***^	0.58 (0.39–0.84)^**^	0.49 (0.30–0.83)^**^
Three negative episodes	0.54 (0.47–0.62)^***^	0.50 (0.38–0.65)^***^	0.49 (0.33–0.73)^***^	0.39 (0.23–0.68)^***^
Four negative episodes	0.52 (0.42–0.66)^***^	0.40 (0.25–0.64)^***^	0.27 (0.12–0.61)^***^	0.13 (0.03–0.56)^**^

^**^P<0.01, ^***^P<0.001.

**Table 4:** Probability of being referred for colposcopy, and prevalence ratio (relative risk) of referral for colposcopy, during the first episode after age of 50 years, by screening history

**Screening history between ages of 40 and 49 years**	**Screening episodes No.**	**Colposcopy referral No. (%)**	**Prevalence ratio of referral (95% CI)**
Negative	42 124	477 (1.18)	0.48 (0.41–0.57)^***^
Inadequate	7056	121 (1.71)	0.73 (0.58–0.92)^**^
Positive	8471	199 (2.35)	1.00

^**^P<0.01, ^***^P<0.001.
